# Long Non-Coding RNAs in Multifactorial Diseases: Another Layer of Complexity

**DOI:** 10.3390/ncrna4020013

**Published:** 2018-05-11

**Authors:** Gabriel A. Cipolla, Jaqueline C. de Oliveira, Amanda Salviano-Silva, Sara C. Lobo-Alves, Debora S. Lemos, Luana C. Oliveira, Tayana S. Jucoski, Carolina Mathias, Gabrielle A. Pedroso, Erika P. Zambalde, Daniela F. Gradia

**Affiliations:** Department of Genetics, Federal University of Parana, Curitiba 81531-980, Brazil; gabriel.cipolla@ufpr.br (G.A.C.); jaqueline.carvalho@ufpr.br (J.C.d.O.); amandasalviano@ufpr.br (A.S.-S.); sara.alves@ufpr.br (S.C.L.-A.); debora.lemos@ufpr.br (D.S.L.); luana.oliveira@ufpr.br (L.C.O.); tayanajucoski@ufpr.br (T.S.J.); carolina.mathias@ufpr.br (C.M.); gabriellearaujop@ufpr.br (G.A.P.); erikazambalde@ufpr.br (E.P.Z.)

**Keywords:** complex diseases, lncRNAs, biomarkers

## Abstract

Multifactorial diseases such as cancer, cardiovascular conditions and neurological, immunological and metabolic disorders are a group of diseases caused by the combination of genetic and environmental factors. High-throughput RNA sequencing (RNA-seq) technologies have revealed that less than 2% of the genome corresponds to protein-coding genes, although most of the human genome is transcribed. The other transcripts include a large variety of non-coding RNAs (ncRNAs), and the continuous generation of RNA-seq data shows that ncRNAs are strongly deregulated and may be important players in pathological processes. A specific class of ncRNAs, the long non-coding RNAs (lncRNAs), has been intensively studied in human diseases. For clinical purposes, lncRNAs may have advantages mainly because of their specificity and differential expression patterns, as well as their ideal qualities for diagnosis and therapeutics. Multifactorial diseases are the major cause of death worldwide and many aspects of their development are not fully understood. Recent data about lncRNAs has improved our knowledge and helped risk assessment and prognosis of these pathologies. This review summarizes the involvement of some lncRNAs in the most common multifactorial diseases, with a focus on those with published functional data.

## 1. Introduction

Multifactorial diseases can be defined as a group of conditions caused by the combination of genetic and environmental factors. Categorized within this group there is a variety of diseases, including cardiovascular conditions, neurological, immunological and metabolic disorders and cancer [1]. From a public health perspective, expenses on multifactorial diseases are at the top of the list of expenditures upon disease treatment in the United States ($2.10 trillion) [2] and are among the leading causes of death worldwide. These data justify the numerous studies focusing on better understanding their molecular basis to improve diagnosis and treatment options.

The Human Genome Project and high-throughput RNA sequencing (RNA-seq) technologies have revealed a surprisingly small number of protein-coding regions in humans. In fact, from our whole genome, less than 2% of the total nucleotide sequence corresponds to protein-coding genes, although most of the human genome is transcribed [3,4,5,6]. The other transcripts include: ribosomal RNA (rRNA); transfer RNA (tRNA); small nuclear RNA (snRNA); small nucleolar RNA (snoRNA); small regulatory RNAs including microRNAs (miRNAs) and piwi-RNAs (piRNA); and long non-coding RNAs (lncRNAs), including here the class of transcribed ultraconserved regions (T-UCRs) [7,8,9,10,11]. The continuous generation of RNA-seq data has made it possible to investigate normal and diseased tissues [12], with the observation that non-coding RNAs (ncRNAs) are strongly deregulated and may be important players in pathological processes [13,14].

From all ncRNA classes, miRNAs and lncRNAs have received major attention [15,16]. miRNAs have been extensively studied in the last decade and a great amount of literature is available, showing their role in many disorders [17,18,19,20].

Unlike miRNAs, lncRNAs comprise a highly diverse group of RNAs [21] with multiple functions and that often exhibit tissue-specific expression patterns [22,23,24]. By definition, lncRNAs are molecules longer than 200 nucleotides that are not translated into functional proteins [25]. Transcriptome studies have revealed that most transcripts belong to the class of lncRNAs [26] and that more molecules are yet to be identified [27]. They are classified into five groups—as sense, antisense, bidirectional, intronic, and intergenic lncRNAs—according to their relative position to a protein-coding gene [28].

In general, RNA molecules can establish specific interactions with DNA, RNA or proteins, by base pairing and/or by their tertiary structure, then forming complexes with distinct biological functions. In the nucleus, lncRNAs act to regulate gene activity through chromatin remodeling and/or by recruiting transcription factors and enhancers. Their association with splicing factors and exportation complexes also interferes with gene regulation, changing the stability and availability of mRNAs in specific circumstances [29]. In the cytoplasm, lncRNAs can act in different ways. They may target mRNA transcripts and modulate their stability, by recruiting proteins that recognize or, conversely, hiding these elements from the degradation machinery. They can also block translation by double stranding with mRNA or even promote cap-independent translation. In this way, long non-coding RNAs can regulate their neighboring genomic environment (in cis) or in distant sites of action (in trans) [30]. Genes of lncRNAs can host miRNA sequences and, in this way, these molecules may be expressed coordinately. Finally, lncRNAs can act as sponges (or competing endogenous RNAs, ceRNAs) of miRNAs and proteins, preventing them from binding to their usual targets [31]. More recently, new studies have shed light on the encoding role of lncRNAs, including the description of functional micropeptides [32,33,34,35].

LncRNAs are essential for both pluripotency in embryonic stem cells [36] and the formation and physiology of specialized cell types [37,38,39]. They are also associated with disease onset and progression, with increasing evidence for their differential expression in a variety of pathological conditions [19,28,40,41,42,43,44,45,46,47,48,49,50]. Among all multifactorial diseases, lncRNAs have already been well described in cancer [9,45,51,52,53,54,55,56,57,58]. Here, we will summarize the involvement of some lncRNAs in cardiovascular, autoimmune and neurodegenerative diseases and in the most common psychiatric disorders. This review focuses on lncRNAs with published functional data.

## 2. Long Non-Coding RNAs in Complexes Diseases

### 2.1. Cardiovascular Diseases

Cardiovascular diseases (CVD) are the most common cause of death worldwide and contribute substantially to health and economic burdens globally. Some factors contribute to cardiovascular disease development such as unhealthy behaviors (smoking, physical inactivity, inappropriate nutrition, obesity, abusive use of alcohol), some health conditions (hypertension, high cholesterol, metabolic syndromes and diabetes mellitus) and genetics factors (alleles, mutation, methylation pattern, chromosomal abnormalities) [59,60].

CVD are a group of disorders that involves heart and blood vessel diseases that are related to atherosclerosis in most cases [60,61]. Atherosclerosis is a chronic disease with inflammatory manifestation in which plaques (made of fatty substances, cholesterol, cellular waste products, fibrin and calcium) build up on walls of arteries making it harder for blood to flow [61]. Therefore, this condition is the primary origin of various cardiovascular diseases: angina, myocardial infarction, heart failure, heart attack, ischemic stroke, coronary artery diseases, and other cardiovascular problems [62].

The main events in atherosclerosis involve lipid metabolism, inflammatory response, cell proliferation and apoptosis, adhesion and migration [63]. This multifactorial process is attributed to the interactions of several key components accompanied by fatty plaque formation, a cascade of inflammatory response, proliferation of smooth muscle cells, and migration of monocytes [64]. Consequently, a cascade of immune-inflammatory responses triggers wound healing and repair.

Among the genetic contribution to CVD, lncRNAs may participate in atherosclerosis regulation but several of them remain functionally uncharacterized [65,66,67,68,69,70,71,72]. LncRNAs influence the transcriptional landscape from epigenetic chromosome modification and remodeling to the regulation of transcriptional and post-transcriptional processes, and regulate the functions of endothelial cells (ECs), smooth muscle cells (SMCs) and macrophages, and vascular inflammation and metabolism, suggesting the involvement of lncRNAs in the manifestation and progression of CVD [73].

The lncRNA ANRIL (CDKN2B antisense RNA 1—CDKN2B-AS1) regulates p16 and p15 expression affecting proliferation, development and progression of atherosclerosis [74,75]. ANRIL can interact with polycomb repressive complex 2 (PRC 2) components such as SUZ12 and CBX7, promoting the H3K27me3, resulting in the epigenetic repression of INK4α. These interactions can influence p14, p15 and p16 expression [75,76,77]. There is evidence showing that polymorphisms in ANRIL can interfere in its interaction with p14, 15, and p16, affecting the expression of these proteins and, in consequence, affecting the PRC complex role, therefore influencing progression to some diseases and contributing, for example, to coronary artery disease (CAD) susceptibility [78,79]. This lncRNA is located in 9p21.3 and a risk allele is carried by 75% of the European population that confers risk for coronary atherosclerosis [80], ischemia stroke [81], intracranial aneurysm [82,83], myocardial infarction [84], abdominal aortic aneurysms [85] and peripheral vascular disease [86]. A meta-analysis study demonstrated that this locus is significantly associated with early onset of heart disease [87].

The myosin heavy chain-associated RNA transcripts, also known as MyHeart (MHRT), is a cardioprotective antisense lncRNA encoded by the murine myosin heavy chain 7 (*MYH7*) and is suggested to have a conserved biological function [65]. BRG1-HDAC-PARP (SMARCA4-HDAC9-PARP1) chromatin complex inhibits *MYH6* and *MYH7* (MHRT) transcription. In the healthy heart, MHRT binds to BRG1 through its helicase domain, preventing BRG1 from repressing its target gene *MYH6* which results in the suppression of cardiac hypertrophy and heart failure [65,88,89]. Increased levels of stress stimuli have been found to downregulate MHRT through the activation of BRG1-HDAC-PARP chromatin repressor complex, which exacerbates cardiac hypertrophy. Conversely, MHRT inhibits BRG1 and increases the levels of genes involved in cardiomyopathy [65,88,90,91]. This lncRNA was also shown to be a novel predictive biomarker of heart failure [92].

Cardiac hypertrophy-associated transcript (CHAST) is a pro-hypertrophic lncRNA that was recently described as a cis-regulator of *PLEKHM1* located on the opposite strand [71]. This lncRNA may have an important role in cardiac remodeling and heart failure despite not being a cardiac-specific lncRNA, but its cell type-specific expression is sensitive to pressure overload pro-hypertrophic stimuli in vitro and in vivo. The *CHAST* gene has a binding site for the pro-hypertrophic NFAT (nuclear factor of activated T cells) transcription factor which negatively regulates PLEKHM1, a protein with autophagy regulatory activity [93]. In this way, CHAST can promote the inhibition of this process and its overexpression can drive cardiomyocytes hypertrophy. This lncRNA was found to be upregulated in a murine model and in patients with aortic stenosis [71,93]. However, the mechanism of CHAST/PLEKHM1 interaction it is still unknown. It seems that CHAST affects pathways of cardiac muscle morphogenesis and Wnt signaling, an additional driver of cardiac hypertrophy, but the mechanism is still unknown [94]. Bioinformatics analysis shows that CHAST can interact with vinculin, laminins and calmodulin, which have already been related to some cardiac diseases [95,96,97]. Inhibition of CHAST shows some therapeutic relevance to prevent or attenuate cardiac remodeling before and after induction of hypertrophy, respectively [71].

LIPCAR (MT-LIPCAR—mitochondrially encoded long non-coding cardiac-associated RNA) is a mitochondrial lncRNA associated with a higher risk of mortality in patients with heart failure [98]. In a study measuring plasma RNAs in patients experiencing heart remodeling following myocardial infarct (MI), and in healthy individuals by a global transcriptomic technology, LIPCAR was detected as downregulated during the initial stages of MI, whereas it was upregulated during the later stages. Patients with chronic heart failure had elevated levels of LIPCAR as compared to patients with continuous heart remodeling at one year after MI. Among a group of 344 individuals with systolic heart failure, LIPCAR levels were indeed associated with future cardiovascular health, suggesting that LIPCAR is a novel biomarker of cardiac remodeling and predicts future death in patients with heart failure [66].

CARMEN (CARMN—Cardiac mesoderm enhancer associated ncRNA) is an enhancer-associated lncRNA that has trans-repressive roles during cardiac differentiation. Ounzain et al. showed, by RNA immunoprecipitation, that this lncRNA interacts with SUZ12 and EZH2, two components of the PRC2, but the molecular mechanisms of this interaction are currently unknown. They found that the inhibition of cardiac specification and differentiation in cardiac precursor cells occurs when CARMEN is knocked down through the ablation of gene expression of cardiac transcription factors and differentiation markers, including GATA4, NKX2.5, TBX5, MYH6, MYH7, and TNNI. During pathological remodeling in the human heart, CARMEN expression is activated and necessary for maintaining cardiac identity in differentiated cardiomyocytes [68].

Cardiac hypertrophy occurs because of a heart damage compensation mechanism, but its molecular basis is still being characterized. Some evidence has demonstrated that myeloid differentiation primary response 88 (*MYD88*) gene has an impact on cardiac pathology [99,100]. This gene was characterized as a target of miR-489, which prevents hypertrophic responses by regulating the expression of *MYD88* [67]. In the same work, Wang and colleagues identified a lncRNA named cardiac hypertrophy related factor (CHRF) that acts as an endogenous sponge for miR-489, regulating cardiac hypertrophy. CHRF induces hypertrophic responses in vitro and increases cardiomyocyte apoptosis in vivo, whereas knockdown attenuates cardiac hypertrophy in the angiotensin II-induced mouse model of heart failure [101].

A combination of increasing expression of genes related to muscle contraction, such as *MYH7* and *ACTA1*, and genes implicated in the control of extracellular fluid volume, such as *ANF*, contribute to the development of cardiac hypertrophy. The lncRNA named CHAER (cardiac-hypertrophy-associated epigenetic regulator) associates with PRC2, preventing H3 lysine 27 methylation at the promoter regions of those genes. Wang et al. demonstrated that CHAER-PRC2 interaction is induced following stress stimulation, and subsequent methylation at the promoter regions of genes involved in cardiac hypertrophy contributes to the development of cardiac hypertrophy and pathological remodeling. The authors also suggest that this interaction could serve as a potential therapeutic target for the treatment of this kind of cardiovascular disease [72].

It has been verified that polymorphisms in the lncRNA MIAT confer risk of myocardial infarct [78]. In a recent publication, the same group demonstrated that MIAT is upregulated in the myocardium of diabetic rats and may act as a ceRNA to increase Dapk2 expression by sponging miR-22-3p, contributing to cardiomyocyte apoptosis [102]. Studies showed that mice that overexpress miR-150 in the heart are resistant to cardiac hypertrophy through downregulation of serum response factor (SRF). Conversely, the loss of function of miR-150 produced the opposite effects [103]. It has been determined that over-expression of Miat in H9C2 cells reduces mir-150 expression, suggesting that this lncRNA acts as a sponge for miR-150 during hypertrophy development [104]. In an AngII-induced cardiac hypertrophy mouse model, lncRNA Miat was significantly increased and necessary for cardiac hypertrophy. In addition, Yan et al. found that lncRNA Miat regulates microvascular dysfunction by functioning as a ceRNA in rat models [105]. The regulation of gene expression by recruiting miRNAs seems to be well characterized for this lncRNA, at least in animal models. In humans, it was demonstrated that MIAT is overexpressed in Chagas disease patients with inflammatory dilated chronic cardiomyopathy, compared to non-inflammatory dilated cardiomyopathy and controls, suggesting that this lncRNA may be associated with the increased severity of this pathology, operating by yet unknown mechanisms [106].

The lncRNA H19 may participate in the progression of CAD since it is expressed in human atherosclerotic plaques and in a rat model of carotid artery injury [107,108], and that hyperhomocysteinemia, an independent risk factor for CAD, could increase the expression of H19 in aorta and vascular smooth muscle cells [109]. H19 is upregulated in pathological cardiac hypertrophy and heart failure [110], and may act through different mechanisms. First, it may protect cardiomyocytes from phenylephrine-induced hypertrophy through miR-675, mapped in the H19 locus. The authors demonstrated that its overexpression upregulates the expression level of miR-675 in cardiomyocytes, which in turn inhibits hypertrophic growth of these cells. Furthermore, a pro-hypertrophic factor, Ca/calmodulin-dependent protein kinase IIδ (CaMKIId), a direct target of miR-675, partially mediated the effect of H19 on cardiomyocyte hypertrophy [69]. Second, the downregulation of H19 inhibits miR-19b, a microRNA that was previously demonstrated to promote cell proliferation and suppress apoptosis of mouse embryonic carcinoma cells, controlling Sox6 expression which, in turn, promotes cell proliferation and inhibits cell apoptosis during late-stage cardiac differentiation [111,112]. Third, the contribution of lncRNA H19 to cardiac fibroblast proliferation and fibrosis can be in part through repression of DUSP5/ERK1/2 [113]. Cardiac fibrosis depends on cardiac fibroblast activation. The main pathway involved in this activation and cell proliferation is MAPK/ERK1/2 and its regulation and repression by DUSP5 [114]. Tao et al. observed that H19 is upregulated in activated cardiac fibroblast and fibrosis tissue as well as p-ERK1/2, although the upregulation of ERK1/2 is often preceded by the dysregulation of DUSP5. So, they investigated the possible relationship between these two phenomena and found that overexpression of H19 is correlated with DUSP5 downregulation and p-ERK1/2 upregulation in cardiac fibroblast [113]. Finally, Pan suggested that H19 regulates atherosclerosis via MAPK and NF-kB signaling pathway inducing cell proliferation and reducing apoptosis of vascular smooth muscle cells [115].

The lncPPARδ has been indicated to be a good biomarker for CAD [116]. LncPPARδ regulates the expression of neighboring protein-coding gene *PPARδ* which in turn acts on its targets ADRP and ANGPTL4. Activation of PPARδ increases cholesterol efflux from the macrophages in the lesions and decreases trans-endothelial migration of leukocyte/monocytes into the arterial wall, thus reducing the atherosclerotic lesion size [117], indicating its important role in the process of CAD development.

Besides its roles in cancer [118,119,120], UCA1 inhibited the expression of miR-1 and protected the cardiomyocyte against H_2_O_2_-induced apoptosis mediated by caspase3 through downregulation of the tumor suppressor gene p27, providing new insights into the roles of lncRNAs in the development of ischemia/reperfusion-induced injury [121]. UCA1 was proposed as a novel biomarker for acute myocardial infarction since its plasma levels are significantly decreased 12 h after myocardial infarction but elevated after 72 h in comparison with healthy controls. This lncRNA may become a classical marker for cardiac injury such as cardiac troponin I or creatine kinase, improving the diagnose [122].

### 2.2. Autoimmune Diseases

Autoimmune diseases are a class of various multifactorial disorders, characterized by the exacerbated response of the immunological system against self-antigens. Several lncRNAs from loci associated with autoimmune diseases were found enriched in immune cell types. Moreover, a co-expression pattern of these lncRNAs with protein-coding genes is suggestive of a common pathway in autoimmunity [123].

The Foxp3 long intergenic non-coding RNA (FLICR) is a lncRNA neighboring the transcription factor *FOXP3*. FLICR negatively regulates FOXP3, decreasing its levels in regulatory T (Treg) cells, mainly in conditions of IL-2 deficiency. By promoting the decreased activity of Treg cells, FLICR is associated with the induction of autoimmunity [124]. Furthermore, the lncRNA GAS5 has been found in microarray analysis to be differentially expressed in the autoimmune diseases rheumatoid arthritis, systemic lupus erythematosus, multiple sclerosis and sarcoidosis, as well as in some infectious diseases, indicating the GAS5 role in immune functions and regulation [125]. LncRNAs involved in some autoimmune diseases are discussed in the next sections.

#### 2.2.1. Rheumatoid Arthritis

Rheumatoid arthritis (RA) is the most common inflammatory arthritis and is a major cause of disability. In RA, CD4^+^ T cells, B cells and macrophages infiltrate the synovium and sometimes organize into discrete lymphoid aggregates with germinal centers [126]. The understanding of RA pathogenesis is still a challenge but may involve complex mechanisms of synovial inflammation and lncRNA dysregulation.

H19 overexpression has been reported in synovial tissue from patients with RA [127]. HOTAIR is another lncRNA that is overexpressed in RA, specifically in peripheral blood mononuclear cells (PBMCs) and exosomes purified from patient’s serum, suggesting that HOTAIR may be used as a potential biomarker for RA diagnosis [128]. The *C5T1lncRNA* (C5-OT1—C5 3’UTR overlapping transcript 1) is transcribed from the region between *TRAF1* and *C5* genes previously associated with RA pathogenesis. It is localized predominantly in the nucleus and its expression correlates with C5 mRNA levels in many tissues [129].

Analysis of T cells from RA patients and controls demonstrated some deregulated lncRNAs, such as increased expression of LOC100652951 and LOC100506036 [130]. Furthermore, an investigation of lncRNA expression in rheumatoid arthritis fibroblast-like synoviocytes (RAFLS) following the administration of quercetin found, among other things, differential expression of MALAT1, HOTAIR, MEG3, CBR3-AS1, GAS5 and YIYA. In this study, MALAT1 was found to be upregulated approximately 4-fold and knockdown of MALAT1 inhibited apoptosis in RAFLS and led to the activation of the PI3K/AKT pathway [131].

#### 2.2.2. Type 1 Diabetes

Type 1 diabetes is a chronic disease in which genetic predisposition, coupled with environmental influence predominantly in early life, induces pancreatic beta cell autoimmunity, eventually resulting in progressive apoptotic destruction of these cells. Some research has been done on the involvement of lncRNAs in diabetes pathogenesis. A microarray analysis of a murine insulin-secreting cell line (MIN6) before and after exposition to proinflammatory cytokines (IL-1β, TNF-α, and IFN-γ) reported many lncRNAs differentially expressed in MIN6, many of which were modified by cytokine treatment: 467 transcripts were upregulated and 219 were downregulated. Four upregulated lncRNAs that displayed the most significant expression changes were evaluated in prediabetic non-obese diabetic (NOD) mice islet cells, where they were also overexpressed. These results allowed the conclusion that lncRNA overexpression sensitizes beta cells to apoptosis, probably contributing to their failure during the initial phases of the disease [132].

In addition, a similar study was performed by Sun et al., who conducted a microarray analysis in MIN6 cells exposed to the same proinflammatory cytokines from the previous study, resulting in 444 upregulated and 279 downregulated lncRNAs. A lncRNA-mRNA co-expression network identified interactions between the differentially expressed lncRNAs and mRNAs. The most enriched pathways among upregulated lncRNAs were: cytokine–cytokine receptor interaction, the NFkB signaling pathway, and toll-like receptor signaling. Regarding the downregulated lncRNAs, the most enriched were: metabolic pathways, viral carcinogenesis and protein processing in the endoplasmic reticulum [133]. Together, these results are indicative of an important role of lncRNAs in cytokine-mediated beta cell dysfunction.

#### 2.2.3. Systemic Lupus Erythematosus

Systemic lupus erythematosus (SLE) is characterized by the production of pathogenic autoantibodies against nuclear autoantigens, pointed as the primary cause of tissue damage. There are diverse and heterogeneous clinical presentations in SLE patients, in which different tissues can be affected. The SLE autoimmunity arises by means of complex mechanisms involving every key facet of the immune system [134]. Purified monocytes from SLE patients and controls evidenced 29 and 60 lncRNAs upregulated and downregulated in SLE, respectively [135]. The expression of four lincRNAs was measured in PBMCs from SLE patients, patients with rheumatoid arthritis and subjects without autoimmune diseases, and it was found that linc0949 and linc0597 expression was significantly decreased in patients with SLE compared to patients with RA and control subjects. Besides, the level of linc0949 was reduced in patients with SLE who had the presence of cumulative organ damage and lupus nephritis. For linc1992 and linc3995, no significant differential expression was found [136]. More recently, the contribution of lncRNA NEAT1 for SLE pathogenesis was investigated. The NEAT1 expression was abnormally increased in monocytes from SLE patients. Additionally, it was demonstrated that NEAT1 expression was induced by lipopolysaccharide via p38 activation. Moreover, NEAT1 silencing experiments with target-specific antisense oligonucleotides resulted in significantly lower expression of a group of chemokines and cytokines, including IL-6 and CXCL10, highlighting the NEAT1 role in the SLE autoimmune response [137].

#### 2.2.4. Psoriasis

Psoriasis is a common inflammatory disease in which skin cells multiply faster than normal, forming scales and red plaques patching the skin. Among the lncRNAs with a role in psoriasis pathogenesis, PRINS (psoriasis-associated non-protein coding RNA induced by stress) is the most well-stablished. PRINS regulates the *G1P3* gene, which has an anti-apoptotic effect and is overexpressed in hyper proliferative lesional and non-lesional psoriatic epidermis. Thus, PRINS deregulation leads to a higher expression of G1P3, which may contribute to psoriasis as a result of keratinocyte proliferation in psoriatic lesion development [138]. In psoriatic patients, the uninvolved epidermis has a higher expression of PRINS, compared both healthy and psoriatic lesioned epidermis. As it is known that PRINS plays a protective role in cellular stress response, this differential expression suggests that PRINS might be involved in the susceptibility to psoriasis, and not in the psoriatic lesion maintenance [139]. In the functional studies of Szegedi et al., the phosphoprotein nucleophosmin (NPM) was found in HaCaT and NHEK cell lysates as a potential PRINS-direct interacting molecule. The authors revealed by immunohistochemistry that in psoriatic-lesioned samples, NPM is overexpressed in dividing cells of the basal layers compared with healthy and non-lesioned samples. It was also found that PRINS prevents the translocation of NPM from the nucleolus to the nucleoplasm of ultraviolet-B (UV-B) irradiated cultured keratinocytes, an incident that occurs in certain cells as a stress response after skin UV-B irradiation [140].

In association studies, the lncRNA PSORS1C3, located in PSORS1 (in 6p21.3) and near to *HLA-C* gene region, was also related to psoriasis susceptibility. The PSORS1C3 alleles −79C, −26C and +246 were strongly associated with psoriasis in a Caucasian population [141], and the allele 582A was associated with psoriasis vulgaris in Chinese patients [142].

Furthermore, several other lncRNAs were found as differentially expressed in psoriatic subjects in studies using large-scale analysis [143,144,145,146], and many of them are co-expressed with immune-related genes [144]. These data demonstrate the involvement of lncRNAs in this disease and raise the question of their role in other autoimmune diseases of the skin.

#### 2.2.5. Other Autoimmune Diseases

Studies on lncRNA roles in other autoimmune diseases have also been done. In Sjogren’s Syndrome, increased expression of TMEVPG1 lncRNA (IFNG-AS1—IFNG antisense RNA 1) was detected in CD4^+^ T lymphocytes and positively correlated with Th1 cell proportion among CD4^+^ T cells [147]. In Kawasaki disease, the lncRNA PINC (pregnancy induced noncoding RNA) was found overexpressed in human umbilical vascular endothelial cells (HUVECs). It was also reported that knockdown of PINC enhanced the viability of HUVECs treated with TNF-α, increased the expression of anti-apoptotic genes and, on the other hand, reduced the expression of apoptotic genes [148]. In addition, THRIL lncRNA expression was significantly lower in Kawasaki disease patients during the acute phase than in the post-treatment convalescent phase [149]. In autoimmune thyroid disease, a single nucleotide variant Ex9b-SNP10 located in the promoter region of B cell-specific lncRNA SAS-ZFAT was associated with increased risk for disease [150]. For celiac disease (CeD), it has been demonstrated that Lnc13 (lncRNA13) regulates a subset of CeD-related inflammatory genes, that may account for inflammation observed, contributing to its pathogenesis [151].

### 2.3. Neurodegenerative Disorders

Neurodegenerative disorders are characterized by neuronal damage and reduction of brain capacity, ability to think, memory and motor coordination [152]. Many of these diseases have an accumulation of misfolded and different toxic proteins, which contribute to neuronal death [153]. In addition to the role of many genes involved in regulation, production, and degradation of these malfunctioning proteins inside the neurons, part of the neurodegeneration is caused by the involvement of other cells and inflammation components [154]. LncRNA deregulation has also been included as part of these processes.

#### 2.3.1. Alzheimer’s Disease

In Alzheimer’s disease (AD), mutations in one of three genes—the amyloid precursor protein (*APP*) gene and the presenilin protein 1 and 2 genes (*PS1* and *PS2*), which encode subunits of gamma-secretase—contribute to the development of an early onset disease [155], and the allele *E4* of the *APOE* gene increases considerably the risk of development of AD [156]. Toxic proteins in AD are the result of APP processing in amyloid beta peptides (Aβ) and hyperphosphorylation of microtubule-associated TAU protein. Aβ peptides are exported and accumulated outside the neuron, interfering with synapses. The accumulation of abnormal TAU protein causes toxic tangles inside the neuron and facilitates cell death [153]. The process of Aβ deposition is connected to the development of AD symptoms and it has been reported that lncRNA regulates this processing pathway.

BACE1 antisense (BACE1-AS) is an lncRNA which regulates its host gene-encoding β-site APP-cleaving enzyme 1 (*BACE1*) [157]. In AD, its upregulation drives an increase of Aβ production, by increasing the stability of BACE1 mRNA through the formation of RNA duplexes. Through the silencing of BACE1-AS in SH-SY5Y human neuroblastoma cells treated with exogenous Aβ, a significant reduction of cytotoxic effect was observed and these cells maintained their normal state, suggesting that BACE1-AS is critical in AD development [158].

Another lncRNA that regulates its host gene is 17A, located in the intron 3 of the *GPR51* gene coding for the GABA B2 receptor, whose mRNA was found upregulated in the brain of AD. 17A expression is induced in response to inflammatory stimuli and is reduced by the treatment with non-steroidal anti-inflammatory drugs, such as diclofenac. They also demonstrated that 17A controls the alternative splicing of GPR51 mRNA; decreases transcription of the canonical form of GABA-B R2, impairing GABA-B signaling; and enhances the secretion of Aβ peptide in SH-SY5Y cells treated with IL-1α [159].

Deregulated cholesterol metabolism is a risk factor for the development of AD, interfering with Aβ production. The lncRNA n336934 is upregulated in AD brains and associated with the cholesterol homeostasis pathway [160]. In the same study, the authors also identified n341006 downregulated in AD patients brain and associated with protein ubiquitination and proteasome system pathway, affecting the toxic Aβ degradation.

Magistri et al. investigated the expression of RNAs in the brain from AD patients and age-matched controls. Four lncRNAs (HAO2-AS, EBF3-AS, AD-linc1 and AD-linc2) were reported as having a differential expression. AD-linc2 was not detected in the normal brain, though this lncRNA was found upregulated in specific regions of the brain (superior frontal gyrus, entorhinal cortex, hippocampus, and cerebellum) of AD patients. The authors investigated if the differential expression was caused by Aβ accumulation. Indeed, neuronal damage and the level of AD-linc1 were increased in cells treated with exogenous Aβ [161].

#### 2.3.2. Parkinson’s Disease

Parkinson’s disease (PD) is characterized by the accumulation of misfolded α-synuclein protein, which forms aggregates called Lewy bodies affecting the neuron viability [162]. Clinically PD is characterized by stilly shacking, bradykinesia, muscular rigidity and abnormal posture [163]. In a few percentage of cases, PD behaves as a monogenic condition, with mutations in one of the genes involved in the accumulation of α-synuclein and coding for components of the ubiquitin-proteasome system: α-synuclein (SNCA/PARK1), leucine-rich repeat kinase 2 (LRRK2/PARK8), ubiquitin carboxyl-terminal esterase L1 (UCHL1), Parkinson protein 2 (Parkin/PARK2), PTEN-induced kinase 1 (PINK1/PARK6), PD 7 (DJ-1/PARK7) and PD 9 (ATP13A2/PARK9) [152,164].

The α-synuclein pathway is regulated by the lncRNA MALAT1, which is highly expressed in neurons and regulates a subset of genes involved in the nervous system development and in α-synuclein production. The level of MALAT1 was increased in SH-SY5Y cells after exposure to MPP (1-methyl-4-phenylpyridinium—that induces neurodegeneration to dopamine neurons) and decreased when treated with the immunosuppressant β-asarone (*cis*-2,4,5-trimethoxy-1-allyl phenyl—a neuroprotective drug). Additionally, MALAT1 overexpression upregulated α-synuclein expression, while inhibition of MALAT1 downregulated its expression, by a suggested mechanism in which MALAT1 bound α-synuclein protein enhancing its stability [165].

Kraus et al. observed lncRNAs (MALAT1, HUC1 and 2, lincRNA-p21, SNHG1 and NEAT1) deregulated in different parts of the brain. HUC 1 and 2 were found downregulated, while the others were upregulated in PD compared with controls. MALAT1, lincRNA-p21 and small nucleolar RNA host gene 1 (SNHG1) may be involved in apoptosis pathways and the molecular pathogenesis of PD may be associated with activation of apoptotic events by the p53 pathway [166].

Another lncRNA that was found to be overexpressed in PD patients is AL049437. The reduction in AL049437 level increased cell viability, mitochondrial transmembrane potential, mitochondrial mass, and tyrosine hydroxylase secretion. The lncRNA AK021630, downregulated in the PD brain, is involved in the same pathways, while its overexpression increases cell survival [167].

Mutations in the multifunctional protein LRRK are the major cause of familial and sporadic PD. LRRK2 may interact with the α-synuclein and tau pathway, contributing to PD development, although the mechanism has not yet been defined [168]. Two lncRNAs located in the LRRK2 locus, UC001lva.4 and AC079630, were found to be deregulated in cerebrospinal fluid (CSF) from patients with PD, however LRRK2 expression was unaltered. The roles of these two lncRNA are still unclear [169]. The lncRNA HOTAIR is also important to the development of PD. In an animal model, both HOTAIR and LRRK2 were upregulated and an overexpression of HOTAIR increased the stability and level of LRRK2 mRNA, while the knockout of HOTAIR enhanced the cell viability in SH-SY5Y cells [170].

#### 2.3.3. Huntington’s Disease

LncRNAs are also studied in Huntington’s disease (HD) models. HD is a progressive brain disorder that causes uncontrolled movements, emotional problems, and loss of thinking ability (cognition) caused by trinucleotide (CAG) repeat expansion in the huntingtin (*HTT*) gene, resulting in a toxic huntingtin protein [153]. The first study with lncRNA in HD was performed by Johnson and colleagues. They investigated the differential expression of REST (RE1-silencing transcription factor) targets, affected by mutant huntingtin and found two lncRNAs (HAR1F and HAR1R), located in the HAR1 locus, downregulated in the striatum of HD patients [171].

In an animal model for HD, the LINC00035, also called ABHD11-AS1 (abhydrolase domain containing 11 antisense) in humans and Abhd11os in mice, was investigated by Francelle and colleagues. They showed that the level of Abhd11os was decreased in HD mouse models, that Abhd11os overexpression protects against neuronal toxicity, and that its knockdown exacerbates the toxicity caused by the mutant huntingtin [172].

#### 2.3.4. Amyotrophic Lateral Sclerosis

Amyotrophic lateral sclerosis (ALS) is a progressive neurodegenerative disease of motor neurons. About 10% of ALS cases are inherited, out of which 20% are caused by mutations in the gene encoding superoxide dismutase 1 (*SOD1*), which aggregates and deposits inside the neuron, causing death [153].

In ALS, NEAT has been found to be predominantly expressed in spinal motor neurons in an early phase of the ALS pathological process. NEAT1 is modestly expressed in the human brain and the transcript NEAT1_2 is barely detectable in the central nervous system, while it was found in the brain of ALS patients. NEAT1_2 acts as a scaffold and changes the functions of TDP-43 and FUS/TLS proteins already associated with ALS and involved in RNA processing and stress granule formation [173].

### 2.4. Psychiatric Disorders

Psychiatric disorders include several mental illnesses which involve psychological and/or neurological disabilities. To date, it is known that certain lncRNAs are involved in the pathophysiology of common psychiatric diseases.

Copy number variations (CNVs), especially deletions upstream the *NRXN1* gene, were identified to affect the lncRNA AK127244, segregating with psychiatric conditions in affected individuals of a family [174]. Furthermore, translocations in the disrupted in schizophrenia (DISC) genome locus, including DISC1 (which encodes the scaffold protein DISC1) and DISC2, a lncRNA antisense to DISC1, have been linked with various psychiatric disorders, such as schizophrenia (SZ), autistic spectrum disorders (ASD), major depression disorder (MDD), among others [174,175]. DISC2 is involved in SZ pathogenesis possibly by regulating DISC1 expression [175,176].

#### 2.4.1. Schizophrenia

Schizophrenia (SZ) is a mental disorder characterized by hallucinations, delusions and loss of reality. Several lncRNAs were found to be deregulated in SZ. In a study with murine cortical neurons, neuronal depolarization was induced, and then transcriptional changes were evaluated at different time points in response to neuronal activity, evidencing that MIAT was the most downregulated lncRNA [177]. MIAT binds to splicing factors QKI and serine/arginine-rich splicing factor 1 (SFSF1), and its inhibition leads to increased levels of splice variants of *DISC1* and *ERBB4*, both upregulated genes in post-mortem schizophrenic brains [178]. Furthermore, in an association study in a Chinese population, the MIAT tag SNPs rs1894720 and rs4274 were associated with paranoid SZ [179].

The 1p21.3 loci have been associated with SZ in genome wide association studies (GWAS). Located in this genomic region, miR-137 and (dihydropyrimidine dehydrogenase (DPYD) have both been previously associated with SZ and neurological and psychiatric disorders [180,181]. The lncRNA linc01930 (EU358092) was also characterized in this SZ-associated region, being co-expressed with miR-137 and acting as a regulatory transcript, with alteration of expression in response to psychoactive drug administration [182].

The pas-lncRNA is a lncRNA antisense to the promoter of *CSMD1*, a well-known gene associated with SZ. Pas-lncRNA expression is co-regulated with CSMD1, specifically in the central nervous system (CNS) tissue, and presents a high expression in peripheral tissues where CSMD1 is downregulated, suggesting a brain-specific promoter activity regulation in CNS and a relevance of these genes in brain function [183].

Some of the symptoms present in the early and acute phases of SZ are depression and anxiety. The presence of these common psychiatric states in SZ is a challenge in disease diagnosis and choice of appropriate treatment, indicating the importance of distinguishing SZ from depression and anxiety. Cui and colleagues (2017) found, in a microarray analysis, lncRNAs NONHSAT089447, NONHSAT021545 and NONHSAT041499 to be aberrantly expressed in SZ patients, besides another nine lncRNAs with also different expression patterns between SZ, depression and anxiety (see topic below), suggesting these lncRNAs as potential biomarkers for the differential diagnosis of these diseases [184]. Accordingly, NONHSAT089447 and NONHSAT041499 were also found to be upregulated in SZ patients in a previous study. Moreover, these lncRNA levels were significantly reduced after psychotropic treatment, and NONHSAT041499 was negatively correlated with better treatment outcomes [185].

#### 2.4.2. Depression and Anxiety Disorders

Major depression disorder (MDD), which refers to symptoms of depression, is a psychiatric condition mainly characterized by sadness, tiredness, guilt and feelings of low self-esteem, among other mental symptoms which vary among MDD patients, such as suicidal thoughts and anxiety conditions. The anxiety disorders include anxiety, phobias, post-traumatic stress and obsessive-compulsive conditions.

As cited in the last topic, some lncRNAs were found as potential biomarkers to distinguish SZ from MDD and anxiety. Among them, the lncRNAs TCONS_00019174, ENST00000566208, NONHSAG045500, ENST00000517573, NONHSAT034045 and NONHSAT142707 were significantly downregulated in MDD patients in comparison with controls, while ENST00000505825, NONHSAG017299 and NONHSAT078768 were upregulated in anxiety patients [184].

Among several genes differentially expressed in the medial prefrontal cortex (mPFC) of a group of fear-conditioned mice, the lncRNA Miat was significantly downregulated. The fear-related anxiety was further enhanced after Miat knockdown. The authors propose that Miat plays a role in anxiety-like behavior modulation through interaction with Bmi1, a key member of the polycomb repressive complex 1, which epigenetically regulates the expression of the schizophrenia-related gene crystallin beta 1 (*Crybb1*) [186].

In a genome-wide study of DNA methylation patterns in the cortical brain tissues of MDD suicidal individuals and non-psychiatric controls, in MDD suicidal samples was identified a significant hypomethylation upstream of the lncRNA gene *PSORS1C3* [187]. Some co-methylated loci modules associated with propensity to suicide, which were enriched in important gene networks related to suicidality and depression, were also found [187]. This might explain the increased risk for depression and suicide in psoriatic patients [188].

In another recent study, the expression of the Linc01108 was found to be significantly higher in MDD patients in comparison with controls, and the rs12526133 SNP present in this gene also associated with the susceptibility to the disease. Moreover, the rs2272260 SNP, present in the lncRNA LINC00998, which is in turn under expressed in patients compared to controls, is also associated to MDD [189].

The role of the brain-derived neurotrophic factor (BDNF) in some psychiatric disorders, such as MDD and SZ, is well stablished [174]. The antisense to the *BDNF* gene is *BDNF-AS*. It was shown that *BDNF-AS* silencing, highly increases *BDNF* expression, upregulating glial-derived neurotrophic factor (GDNF) and ephrin receptor B2 (EPHB2) and, then, neuronal outgrowth and differentiation [190]. Moreover, it is suggested that BDNF-AS may inhibit, by complementarity, the binding between the 3’UTR of BDNF and miR-124a, a miRNA associated with MDD and neurobehavioral deficits [191,192].

Among the 2007 MDD-differentially expressed lncRNAs found by Liu et al. in a microarray analysis, the lncRNAs located at chr10:874695-874794, chr10:75873456-75873642, and chr3:47048304-47048512 were found to be able to regulate four differentially expressed mRNAs [193]. Moreover, the lncRNAs BACE-AS [157] and BC200 [194] also seem to be involved in depression [174].

#### 2.4.3. Autistic Spectrum Disorders

The autistic spectrum disorders (ASD), which include autism, Asperger’s syndrome and other mental-related diseases, are common mental illnesses diagnosed in childhood, which involve impairments in social interaction and in communication, intellectual disabilities and repetitive behaviors.

In the cluster 5p14.1, to where important common variants associated with ASD map, the moesin pseudogene 1 (*MSNP1*) and its antisense lncRNA *MSNP1-AS* may also be found. MSNP1-AS overexpression was found in post-mortem ASD cerebral cortex and in individuals with the rs4307059 ASD risk allele [195,196]. The overexpression of this lncRNA decreased neurite number and length in human neuronal progenitor cells and dysregulated the expression of genes involved in protein synthesis and chromatin remodeling. MSNP1AS knockdown altered the expression of several genes, mainly those involved in chromatin remodeling and immune response [196].

In a study with a 3-year-old male with ASD, a 2Mb deletion in 1q42 was reported. This genomic region contains the coding genes translin-associated factor X (TSNAX) and DISC1, and the lncRNA DISC2, all of them previously associated with psychiatric disorders. Authors suggest that DISC2 haploinsufficiency may lead to a further dysregulation of the remaining DISC1 in ASD patients [197]. Moreover, in another family study, some deletions in PTCHD1AS1 and PTCHD1AS2, antisense lncRNAs which seem to regulate its overlapping coding gene *PTCHD1*, were associated with ASD in males [198]. Furthermore, analysis of post-mortem prefrontal cortex and cerebellum tissue of ASD patients indicated some lncRNAs as differentially expressed and located around the human imprinted loci C9orf85, SLC4A2, and UBE3A. The *UBE3A* gene is related with Angelman syndrome, a disorder with some ASD symptoms [199]. LncRNAs antisense to *BDNF* (BDNF-AS) and *SHANK2* (SHANK2-AS), as some lncRNAs located within the *HOX* loci, were also associated with ASD [200].

## 3. Conclusions

The discovery of lncRNAs as another intricate layer in multifactorial disease development has generated more questions than answers since they are required for the proper function of higher eukaryote metabolism. Here we summarize the most relevant lncRNAs described until now, focused on those with published functional data (Table 1). The databases of lncRNA sequences in the human genome are still under construction and complete annotation in different cells and tissues is necessary for subsequent work. For clinical purposes, lncRNAs may have advantages mainly because of their specificity and differential expression patterns, as well as their ideal qualities for diagnosis and therapeutics. Besides, the potential to detect distinct circulating lncRNAs between patients and healthy individuals may allow the development of new diagnostic inputs, increasing the accuracy of the tests already available and helping risk assessment and prognosis.

## Figures and Tables

**Table 1:** List of the most relevant lncRNAs and the associated mechanisms.

LncRNA	Expression	Disease	Mechanism	Reference
ANRIL	Down	Coronary Artery Disease (CAD), Myocardial Infarction	Chromatin remodelling (interacts with PRC1 and PRC2); regulation of CDKN2A and CDKN2B expression.	[74,75,76,77,79]
MHRT	Up	Heart failure, Acute Myocardial Infarction, Hypertrophy, Cardiomyopathy	Chromatin remodelling (cardiac stress); interacts with BRG1 preventing its ligation to targets.	[65,88,89,90,91,92]
CHAST	Up	Heart failure, Aortic Stenosis, Cardiac Hypertrophy.	Cardiac remodelling; negatively regulates Plekhm1.	[71,93,94,95,96,97]
LIPCAR	Down	Initial Myocardial Infarction, Chronic Cardiovascular Disease	Cardiac remodeling; unknown mechanism.	[66,98]
CARMEN	Up	Cardiovascular Disease (Cardiac Hypertrophy)	Chromatin remodelling; interacts with SUZ12 and EZH2 (PRC2).	[68]
CHRF	Up	Cardiac Hypertrophy	Sponge for mir-489.	[67,101]
CHAER (mouse)	Up	Cardiac Hypertrophy	Epigenetic Regulator; inhibits H3L27 methylation.	[72]
MIAT	Up	Cardiac Hypertrophy, Myocardial Infarction	Sponge for mir-150.	[78,102,103,104,105,106]
H19	Up	Atherosclerosis, Heart failure, Cardiac Hypertrophy	Epigenetic Regulator; suppresses miR-675 and miR-19b; repression of DUSP5/ERK1/2; regulating MAPK and NF-kB signalling pathway.	[69,107,108,109,110,111,112,113,114,115]
LncPPARδ	Up	Atherosclerosis, Cardiovascular Disease	Decreases cholesterol efflux and increases migration of leukocyte/monocytes into the arterial wall.	[116]
UCA1	Down/Up (Biphasic)	Myocardial Infarction (after 12 h/72 h)	Unknown.	[122]
H19	Up	Rheumatoid Arthritis	Unknown.	[127]
HOTAIR	Up	Rheumatoid Arthritis	Unknown.	[128]
C5T1lncRNA	Up	Rheumatoid Arthritis	Regulates C5 mRNA expression.	[129]
LOC10065295/LOC100506036	Up	Rheumatoid Arthritis	Unknown.	[130]
MALAT 1	Up	Rheumatoid Arthritis	Promotes the apoptosis of rheumatoid arthritis fibroblast-like synoviocytes and leads to the activation of the PI3K/AKT pathway.	[131]
NEAT1	Up	Systemic Lupus Erythematosus (SLE)	Contributes to expression of a group of chemokines and cytokines, including IL-6 and CXCL10.	[137]
TMEVPG1	Up	Sjogren’s Syndrome	Positively correlates expression with Th1 cell proportion among CD4+ T cells.	[147]
PINC	Up	Kawasaki disease	Promotes expression of apoptosis genes in human umbilical vascular endothelial cells (HUVEC).	[148]
PRINS	Up	Psoriasis	Interacts with NPM protein, which is associated with keratinocytes proliferation.	[139]
*BACE1-AS*	Up	Alzheimer’s Disease	Increases the stability of BACE1 mRNA. Aβ production.	[157,158]
*17A*	Up	Alzheimer’s Disease	Alternative splicing of GPR51 mRNA, reducing canonical form of GABAB R2 and impairing GABAB signaling. Aβ production.	[159]
*HAO2-AS, EBF3-AS, AD-linc1 and AD-linc2*	Up	Alzheimer’s Disease	Neurotoxicity.	[161]
*n341006*	Down	Alzheimer’s Disease	Acting in the ubiquitin-proteasome system (UPS), affecting the turnover and degradation, is impaired in AD.	[160]
*n336934*	Up	Alzheimer’s Disease	Cholesterol pathway.	[160]
*MALAT1*	Up	Parkinson’s Disease	α-synuclein production.	[165]
*HOTAIR*	Up	Parkinson’s Disease	Increasing the stability and level of LRRK2 mRNA.	[170]
*AL049437*	Up	Parkinson’s Disease	Apoptosis pathway.	[167]
*AK021630*	Down	Parkinson’s Disease	Apoptosis pathway.	[167]
*p21*	Up	Parkinson’s Disease	Targets p53 and H1F1 (apoptosis pathway).	[166]
*SNHG1*	Up	Parkinson’s Disease	Affects the p53 stability (cellular proliferation).	[166]
*HAR1F and HAR1R*	Down	Huntington’s Disease	Unknown.	[171]
*ABHD11-AS1*	Down	Huntington’s Disease	Affects the neuronal toxicity.	[172]
*NEAT1_2*	Up	Amyotrophic Lateral Sclerosis (ALS)	Changes the TDP-43 and FUS/TLS functions, stress granules formation).	[173]
*DISC2*	Down	Schizophrenia, Autistic Spectrum Disorders (ASD)	*DISC2* regulates *DISC1* expression. Translocation in both genes are involved with psychiatric disorders.	[174,175,176]
*EU358092*	?	Schizophrenia	Located in SZ- associated region 1p21.3 and co-expressed with mir-137; alters response to psychoactive drugs.	[182]
*MIAT*	Down	Schizophrenia, Fear-related anxiety	Increases pathogenic splice variants of *DISC1* and ERBB4. Associated with risk SNPs rs1894720 and rs4274/Possibly interacts with BMI1, regulating Crybb1 expression.	[177,178,179]
*BDNF-AS*	?	Depression	Repress BDNF expression, and neuronal outgrowth and differentiation.	[191,192]
*MSNP1AS*	Up	ASD	Decreases neurite number and length in neuronal progenitor cells; dysregulates the expression of genes involved in protein synthesis and chromatin remodelling.	[196]

The group of diseases are classified by colors: red for cardiovascular; yellow for autoimmune and green for neurological diseases; blue for psychiatric disorders. Up and Down are related to expression levels compared with healthy patients. A question mark means “information not known”.

## References

[1] Hunter D.J. (2005). Gene-environment interactions in human diseases. Nat. Rev. Genet..

[2] Dieleman J.L., Baral R., Birger M., Bui A.L., Bulchis A., Chapin A., Hamavid H., Horst C., Johnson E.K., Joseph J. (2016). US spending on personal health care and public health, 1996–2013. JAMA.

[3] Baird P. (2001). The Human Genome Project, genetics and health. Community Genet..

[4] Consortium I.H.G.S. (2004). Finishing the euchromatic sequence of the human genome. Nature.

[5] Carninci P., Kasukawa T., Katayama S., Gough J., Frith M.C., Maeda N., Oyama R., Ravasi T., Lenhard B., Wells C. (2005). The transcriptional landscape of the mammalian genome. Science.

[6] Birney E., Stamatoyannopoulos J.A., Dutta A., Guigó R., Gingeras T.R., Margulies E.H., Weng Z., Snyder M., Dermitzakis E.T., Thurman R.E. (2007). Identification and analysis of functional elements in 1% of the human genome by the ENCODE pilot project. Nature.

[7] Irminger-Finger I., Kargul J., Laurent G.J. (2014). Non-coding RNAs: A novel level of genome complexity. Int. J. Biochem. Cell Biol..

[8] St Laurent G., Wahlestedt C., Kapranov P. (2015). The Landscape of long noncoding RNA classification. Trends Genet..

[9] Liz J., Esteller M. (2016). lncRNAs and microRNAs with a role in cancer development. Biochim. Biophys. Acta.

[10] Bejerano G., Pheasant M., Makunin I., Stephen S., Kent W.J., Mattick J.S., Haussler D. (2004). Ultraconserved elements in the human genome. Science.

[11] Baira E., Greshock J., Coukos G., Zhang L. (2008). Ultraconserved elements: Genomics, function and disease. RNA Biol..

[12] Costa V., Aprile M., Esposito R., Ciccodicola A. (2013). RNA-Seq and human complex diseases: Recent accomplishments and future perspectives. Eur. J. Hum. Genet..

[13] De Almeida R.A., Fraczek M.G., Parker S., Delneri D., O’Keefe R.T. (2016). Non-coding RNAs and disease: The classical ncRNAs make a comeback. Biochem. Soc. Trans..

[14] Calin G.A., Liu C.G., Ferracin M., Hyslop T., Spizzo R., Sevignani C., Fabbri M., Cimmino A., Lee E.J., Wojcik S.E. (2007). Ultraconserved regions encoding ncRNAs are altered in human leukemias and carcinomas. Cancer Cell.

[15] Geisler S., Coller J. (2013). RNA in unexpected places: Long non-coding RNA functions in diverse cellular contexts. Nat. Rev. Mol. Cell Biol..

[16] Beermann J., Piccoli M.T., Viereck J., Thum T. (2016). Non-coding RNAs in development and disease: background, mechanisms, and therapeutic approaches. Physiol. Rev..

[17] Sethupathy P., Collins F.S. (2008). MicroRNA target site polymorphisms and human disease. Trends Genet..

[18] Ceman S., Saugstad J. (2011). MicroRNAs: Meta-controllers of gene expression in synaptic activity emerge as genetic and diagnostic markers of human disease. Pharmacol. Ther..

[19] Esteller M. (2011). Non-coding RNAs in human disease. Nat. Rev. Genet..

[20] Welten S.M., Goossens E.A., Quax P.H., Nossent A.Y. (2016). The multifactorial nature of microRNAs in vascular remodelling. Cardiovasc. Res..

[21] Ulitsky I., Bartel D.P. (2013). lincRNAs: Genomics, evolution, and mechanisms. Cell.

[22] Clark M.B., Mercer T.R., Bussotti G., Leonardi T., Haynes K.R., Crawford J., Brunck M.E., Cao K.A., Thomas G.P., Chen W.Y. (2015). Quantitative gene profiling of long noncoding RNAs with targeted RNA sequencing. Nat. Methods.

[23] Jiang X., Ning Q. (2015). The emerging roles of long noncoding RNAs in common cardiovascular diseases. Hypertens. Res..

[24] Salviano-Silva A., Lobo-Alves S.C., Almeida R.C., Malheiros D., Petzl-Erler M.L. (2018). Besides pathology: Long non-coding RNA in cell and tissue homeostasis. Non-Coding RNA.

[25] Cabili M.N., Trapnell C., Goff L., Koziol M., Tazon-Vega B., Regev A., Rinn J.L. (2011). Integrative annotation of human large intergenic noncoding RNAs reveals global properties and specific subclasses. Genes Dev..

[26] Hangauer M.J., Vaughn I.W., McManus M.T. (2013). Pervasive transcription of the human genome produces thousands of previously unidentified long intergenic noncoding RNAs. PLoS Genet..

[27] Iyer M.K., Niknafs Y.S., Malik R., Singhal U., Sahu A., Hosono Y., Barrette T.R., Prensner J.R., Evans J.R., Zhao S. (2015). The landscape of long noncoding RNAs in the human transcriptome. Nat. Genet..

[28] Ma Y., Ma W., Huang L., Feng D., Cai B. (2015). Long non-coding RNAs, a new important regulator of cardiovascular physiology and pathology. Int. J. Cardiol..

[29] Schmitz S.U., Grote P., Herrmann B.G. (2016). Mechanisms of long noncoding RNA function in development and disease. Cell. Mol. Life Sci..

[30] Quinn J.J., Chang H.Y. (2016). Unique features of long non-coding RNA biogenesis and function. Nat. Rev. Genet..

[31] Rashid F., Shah A., Shan G. (2016). Long non-coding RNAs in the cytoplasm. Genom. Proteom. Bioinform..

[32] Anderson D.M., Anderson K.M., Chang C.L., Makarewich C.A., Nelson B.R., McAnally J.R., Kasaragod P., Shelton J.M., Liou J., Bassel-Duby R. (2015). A micropeptide encoded by a putative long noncoding RNA regulates muscle performance. Cell.

[33] Nelson B.R., Makarewich C.A., Anderson D.M., Winders B.R., Troupes C.D., Wu F., Reese A.L., McAnally J.R., Chen X., Kavalali E.T. (2016). A peptide encoded by a transcript annotated as long noncoding RNA enhances SERCA activity in muscle. Science.

[34] D'Lima N.G., Ma J., Winkler L., Chu Q., Loh K.H., Corpuz E.O., Budnik B.A., Lykke-Andersen J., Saghatelian A., Slavoff S.A. (2017). A human microprotein that interacts with the mRNA decapping complex. Nat. Chem. Biol..

[35] Matsumoto A., Pasut A., Matsumoto M., Yamashita R., Fung J., Monteleone E., Saghatelian A., Nakayama K.I., Clohessy J.G., Pandolfi P.P. (2017). mTORC1 and muscle regeneration are regulated by the LINC00961-encoded SPAR polypeptide. Nature.

[36] Guttman M., Donaghey J., Carey B.W., Garber M., Grenier J.K., Munson G., Young G., Lucas A.B., Ach R., Bruhn L. (2011). lincRNAs act in the circuitry controlling pluripotency and differentiation. Nature.

[37] Hacisuleyman E., Goff L.A., Trapnell C., Williams A., Henao-Mejia J., Sun L., McClanahan P., Hendrickson D.G., Sauvageau M., Kelley D.R. (2014). Topological organization of multichromosomal regions by the long intergenic noncoding RNA Firre. Nat. Struct. Mol. Biol..

[38] Ramos A.D., Andersen R.E., Liu S.J., Nowakowski T.J., Hong S.J., Gertz C.C., Salinas R.D., Zarabi H., Kriegstein A.R., Lim D.A. (2015). The long noncoding RNA Pnky regulates neuronal differentiation of embryonic and postnatal neural stem cells. Cell Stem Cell.

[39] Dallagiovanna B., Pereira I.T., Origa-Alves A.C., Shigunov P., Naya H., Spangenberg L. (2017). lncRNAs are associated with polysomes during adipose-derived stem cell differentiation. Gene.

[40] Okazaki Y., Furuno M., Kasukawa T., Adachi J., Bono H., Kondo S., Nikaido I., Osato N., Saito R., Suzuki H. (2002). Analysis of the mouse transcriptome based on functional annotation of 60,770 full-length cDNAs. Nature.

[41] Harries L.W. (2012). Long non-coding RNAs and human disease. Biochem. Soc. Trans..

[42] Chen J., Wang R., Zhang K., Chen L.B. (2014). Long non-coding RNAs in non-small cell lung cancer as biomarkers and therapeutic targets. J. Cell. Mol. Med..

[43] Smolle M., Uranitsch S., Gerger A., Pichler M., Haybaeck J. (2014). Current status of long non-coding RNAs in human cancer with specific focus on colorectal cancer. Int. J. Mol. Sci..

[44] Walsh A.L., Tuzova A.V., Bolton E.M., Lynch T.H., Perry A.S. (2014). Long noncoding RNAs and prostate carcinogenesis: The missing ‘linc’?. Trends Mol. Med..

[45] Yarmishyn A.A., Kurochkin I.V. (2015). Long noncoding RNAs: A potential novel class of cancer biomarkers. Front. Genet..

[46] Li T., Mo X., Fu L., Xiao B., Guo J. (2016). Molecular mechanisms of long noncoding RNAs on gastric cancer. Oncotarget.

[47] Mohankumar S., Patel T. (2016). Extracellular vesicle long noncoding RNA as potential biomarkers of liver cancer. Brief. Funct. Genom..

[48] Hon C.C., Ramilowski J.A., Harshbarger J., Bertin N., Rackham O.J., Gough J., Denisenko E., Schmeier S., Poulsen T.M., Severin J. (2017). An atlas of human long non-coding RNAs with accurate 5’ ends. Nature.

[49] Jain S., Thakkar N., Chhatai J., Pal Bhadra M., Bhadra U. (2017). Long non-coding RNA: Functional agent for disease traits. RNA Biol..

[50] Viereck J., Thum T. (2017). Circulating noncoding RNAs as biomarkers of cardiovascular disease and injury. Circ. Res..

[51] Huang T., Alvarez A., Hu B., Cheng S.Y. (2013). Noncoding RNAs in cancer and cancer stem cells. Chin. J. Cancer.

[52] Eades G., Zhang Y.S., Li Q.L., Xia J.X., Yao Y., Zhou Q. (2014). Long non-coding RNAs in stem cells and cancer. World J. Clin. Oncol..

[53] Yang G., Lu X., Yuan L. (2014). LncRNA: A link between RNA and cancer. Biochim. Biophys. Acta.

[54] Fatima R., Akhade V.S., Pal D., Rao S.M. (2015). Long noncoding RNAs in development and cancer: Potential biomarkers and therapeutic targets. Mol. Cell. Ther..

[55] Zhang R., Xia L.Q., Lu W.W., Zhang J., Zhu J.S. (2016). LncRNAs and cancer. Oncol. Lett..

[56] Bhan A., Soleimani M., Mandal S.S. (2017). Long noncoding RNA and cancer: A new paradigm. Cancer Res..

[57] Rao A.K.D.M., Rajkumar T., Mani S. (2017). Perspectives of long non-coding RNAs in cancer. Mol. Biol. Rep..

[58] Camacho C.V., Choudhari R., Gadad S.S. (2018). Long noncoding RNAs and cancer, an overview. Steroids.

[59] Mozaffarian D., Benjamin E.J., Go A.S., Arnett D.K., Blaha M.J., Cushman M., Das S.R., de Ferranti S., Després J.P., Fullerton H.J. (2016). Heart disease and stroke statistics—2016 update: A report from the American Heart Association. Circulation.

[60] World Health Organization (2009). International Statistical Classification of Diseases and Related Health Problems.

[61] American Heart Association My Life Check—Life’s Simple 7. http://www.heart.org/HEARTORG/Conditions/My-Life-Check---Lifes-Simple-7_UCM_471453_Article.jsp#.WvFmGoiFPIV.

[62] Logue J., Murray H.M., Welsh P., Shepherd J., Packard C., Macfarlane P., Cobbe S., Ford I., Sattar N. (2011). Obesity is associated with fatal coronary heart disease independently of traditional risk factors and deprivation. Heart.

[63] Weber C., Noels H. (2011). Atherosclerosis: Current pathogenesis and therapeutic options. Nat. Med..

[64] Glass C.K., Witztum J.L. (2001). Atherosclerosis. the road ahead. Cell.

[65] Han P., Li W., Lin C.H., Yang J., Shang C., Nuernberg S.T., Jin K.K., Xu W., Lin C.Y., Lin C.J. (2014). A long noncoding RNA protects the heart from pathological hypertrophy. Nature.

[66] Kumarswamy R., Bauters C., Volkmann I., Maury F., Fetisch J., Holzmann A., Lemesle G., de Groote P., Pinet F., Thum T. (2014). Circulating long noncoding RNA, LIPCAR, predicts survival in patients with heart failure. Circ. Res..

[67] Wang K., Liu F., Zhou L.Y., Long B., Yuan S.M., Wang Y., Liu C.Y., Sun T., Zhang X.J., Li P.F. (2014). The long noncoding RNA CHRF regulates cardiac hypertrophy by targeting miR-489. Circ. Res..

[68] Ounzain S., Micheletti R., Arnan C., Plaisance I., Cecchi D., Schroen B., Reverter F., Alexanian M., Gonzales C., Ng S.Y. (2015). CARMEN, a human super enhancer-associated long noncoding RNA controlling cardiac specification, differentiation and homeostasis. J. Mol. Cell. Cardiol..

[69] Liu L., An X., Li Z., Song Y., Li L., Zuo S., Liu N., Yang G., Wang H., Cheng X. (2016). The H19 long noncoding RNA is a novel negative regulator of cardiomyocyte hypertrophy. Cardiovasc. Res..

[70] Tang S.S., Cheng J., Cai M.Y., Yang X.L., Liu X.G., Zheng B.Y., Xiong X.D. (2016). Association of lincRNA-p21 haplotype with coronary artery disease in a Chinese Han population. Dis. Mark..

[71] Viereck J., Kumarswamy R., Foinquinos A., Xiao K., Avramopoulos P., Kunz M., Dittrich M., Maetzig T., Zimmer K., Remke J. (2016). Long noncoding RNA Chast promotes cardiac remodeling. Sci. Transl. Med..

[72] Wang Z., Zhang X.J., Ji Y.X., Zhang P., Deng K.Q., Gong J., Ren S., Wang X., Chen I., Wang H. (2016). The long noncoding RNA Chaer defines an epigenetic checkpoint in cardiac hypertrophy. Nat. Med..

[73] Aryal B., Rotllan N., Fernández-Hernando C. (2014). Noncoding RNAs and atherosclerosis. Curr. Atheroscler. Rep..

[74] Burd C.E., Jeck W.R., Liu Y., Sanoff H.K., Wang Z., Sharpless N.E. (2010). Expression of linear and novel circular forms of an INK4/ARF-associated non-coding RNA correlates with atherosclerosis risk. PLoS Genet..

[75] Yap K.L., Li S., Muñoz-Cabello A.M., Raguz S., Zeng L., Mujtaba S., Gil J., Walsh M.J., Zhou M.M. (2010). Molecular interplay of the noncoding RNA ANRIL and methylated histone H3 lysine 27 by polycomb CBX7 in transcriptional silencing of INK4a. Mol. Cell.

[76] Aguilo F., Zhou M.M., Walsh M.J. (2011). Long noncoding RNA, polycomb, and the ghosts haunting INK4b-ARF-INK4a expression. Cancer Res..

[77] Kotake Y., Nakagawa T., Kitagawa K., Suzuki S., Liu N., Kitagawa M., Xiong Y. (2011). Long non-coding RNA ANRIL is required for the PRC2 recruitment to and silencing of p15(*INK4B*) tumor suppressor gene. Oncogene.

[78] Ishii N., Ozaki K., Sato H., Mizuno H., Saito S., Takahashi A., Miyamoto Y., Ikegawa S., Kamatani N., Hori M. (2006). Identification of a novel non-coding RNA, MIAT, that confers risk of myocardial infarction. J. Hum. Genet..

[79] Congrains A., Kamide K., Katsuya T., Yasuda O., Oguro R., Yamamoto K., Ohishi M., Rakugi H. (2012). CVD-associated non-coding RNA, ANRIL, modulates expression of atherogenic pathways in VSMC. Biochem Biophys. Res. Commun..

[80] Ye S., Willeit J., Kronenberg F., Xu Q., Kiechl S. (2008). Association of genetic variation on chromosome 9p21 with susceptibility and progression of atherosclerosis: A population-based, prospective study. J. Am. Coll. Cardiol..

[81] Anderson C.D., Biffi A., Rost N.S., Cortellini L., Furie K.L., Rosand J. (2010). Chromosome 9p21 in ischemic stroke: Population structure and meta-analysis. Stroke.

[82] Bilguvar K., Yasuno K., Niemelä M., Ruigrok Y.M., von Und Zu Fraunberg M., van Duijn C.M., van den Berg L.H., Mane S., Mason C.E., Choi M. (2008). Susceptibility loci for intracranial aneurysm in European and Japanese populations. Nat. Genet..

[83] Yasuno K., Bilguvar K., Bijlenga P., Low S.K., Krischek B., Auburger G., Simon M., Krex D., Arlier Z., Nayak N. (2010). Genome-wide association study of intracranial aneurysm identifies three new risk loci. Nat. Genet..

[84] Helgadottir A., Thorleifsson G., Manolescu A., Gretarsdottir S., Blondal T., Jonasdottir A., Sigurdsson A., Baker A., Palsson A., Masson G. (2007). A common variant on chromosome 9p21 affects the risk of myocardial infarction. Science.

[85] Helgadottir A., Thorleifsson G., Magnusson K.P., Grétarsdottir S., Steinthorsdottir V., Manolescu A., Jones G.T., Rinkel G.J., Blankensteijn J.D., Ronkainen A. (2008). The same sequence variant on 9p21 associates with myocardial infarction, abdominal aortic aneurysm and intracranial aneurysm. Nat. Genet..

[86] Cluett C., McDermott M.M., Guralnik J., Ferrucci L., Bandinelli S., Miljkovic I., Zmuda J.M., Li R., Tranah G., Harris T. (2009). The 9p21 myocardial infarction risk allele increases risk of peripheral artery disease in older people. Circ. Cardiovasc. Genet..

[87] Palomaki G.E., Melillo S., Bradley L.A. (2010). Association between 9p21 genomic markers and heart disease: A meta-analysis. JAMA.

[88] Archer K., Broskova Z., Bayoumi A.S., Teoh J.P., Davila A., Tang Y., Su H., Kim I.M. (2015). Long Non-coding RNAs as master regulators in cardiovascular diseases. Int. J. Mol. Sci..

[89] Poller W., Dimmeler S., Heymans S., Zeller T., Haas J., Karakas M., Leistner D.M., Jakob P., Nakagawa S., Blankenberg S. (2017). Non-coding RNAs in cardiovascular diseases: Diagnostic and therapeutic perspectives. Eur. Heart J..

[90] Wu C., Arora P. (2015). Long noncoding Mhrt RNA: Molecular crowbar unravel insights into heart failure treatment. Circ. Cardiovasc. Genet..

[91] Shen S., Jiang H., Bei Y., Xiao J., Li X. (2017). Long non-coding RNAs in cardiac remodeling. Cell. Physiol. Biochem..

[92] Xuan L., Sun L., Zhang Y., Huang Y., Hou Y., Li Q., Guo Y., Feng B., Cui L., Wang X. (2017). Circulating long non-coding RNAs NRON and MHRT as novel predictive biomarkers of heart failure. J. Cell. Mol. Med..

[93] McEwan D.G., Popovic D., Gubas A., Terawaki S., Suzuki H., Stadel D., Coxon F.P., Miranda de Stegmann D., Bhogaraju S., Maddi K. (2015). PLEKHM1 regulates autophagosome-lysosome fusion through HOPS complex and LC3/GABARAP proteins. Mol. Cell.

[94] Dawson K., Aflaki M., Nattel S. (2013). Role of the Wnt-Frizzled system in cardiac pathophysiology: A rapidly developing, poorly understood area with enormous potential. J. Physiol..

[95] Yamaguchi N., Takahashi N., Xu L., Smithies O., Meissner G. (2007). Early cardiac hypertrophy in mice with impaired calmodulin regulation of cardiac muscle Ca release channel. J. Clin. Investig..

[96] Zemljic-Harpf A.E., Miller J.C., Henderson S.A., Wright A.T., Manso A.M., Elsherif L., Dalton N.D., Thor A.K., Perkins G.A., McCulloch A.D. (2007). Cardiac-myocyte-specific excision of the vinculin gene disrupts cellular junctions, causing sudden death or dilated cardiomyopathy. Mol. Cell. Biol..

[97] Lu J.T., Muchir A., Nagy P.L., Worman H.J. (2011). LMNA cardiomyopathy: Cell biology and genetics meet clinical medicine. Dis. Model. Mech..

[98] Hullinger T.G., Montgomery R.L., Seto A.G., Dickinson B.A., Semus H.M., Lynch J.M., Dalby C.M., Robinson K., Stack C., Latimer P.A. (2012). Inhibition of miR-15 protects against cardiac ischemic injury. Circ. Res..

[99] Ha T., Hua F., Li Y., Ma J., Gao X., Kelley J., Zhao A., Haddad G.E., Williams D.L., Browder I.W. (2006). Blockade of MyD88 attenuates cardiac hypertrophy and decreases cardiac myocyte apoptosis in pressure overload-induced cardiac hypertrophy in vivo. Am. J. Physiol. Heart Circ. Physiol..

[100] Li Y., Si R., Feng Y., Chen H.H., Zou L., Wang E., Zhang M., Warren H.S., Sosnovik D.E., Chao W. (2011). Myocardial ischemia activates an injurious innate immune signaling via cardiac heat shock protein 60 and Toll-like receptor 4. J. Biol. Chem..

[101] Dangwal S., Schimmel K., Foinquinos A., Xiao K., Thum T. (2017). Noncoding RNAs in Heart Failure. Handb. Exp. Pharmacol..

[102] Zhou X., Zhang W., Jin M., Chen J., Xu W., Kong X. (2017). lncRNA MIAT functions as a competing endogenous RNA to upregulate DAPK2 by sponging miR-22-3p in diabetic cardiomyopathy. Cell Death Dis..

[103] Liu W., Liu Y., Zhang Y., Zhu X., Zhang R., Guan L., Tang Q., Jiang H., Huang C., Huang H. (2015). MicroRNA-150 protects against pressure overload-induced cardiac hypertrophy. J. Cell. Biochem..

[104] Zhu X.H., Yuan Y.X., Rao S.L., Wang P. (2016). LncRNA MIAT enhances cardiac hypertrophy partly through sponging miR-150. Eur. Rev. Med. Pharmacol. Sci..

[105] Yan B., Yao J., Liu J.Y., Li X.M., Wang X.Q., Li Y.J., Tao Z.F., Song Y.C., Chen Q., Jiang Q. (2015). lncRNA-MIAT regulates microvascular dysfunction by functioning as a competing endogenous RNA. Circ. Res..

[106] Frade A.F., Laugier L., Ferreira L.R., Baron M.A., Benvenuti L.A., Teixeira P.C., Navarro I.C., Cabantous S., Ferreira F.M., da Silva Cândido D. (2016). Myocardial infarction-associated transcript, a long noncoding RNA, is overexpressed during dilated cardiomyopathy due to chronic Chagas disease. J. Infect. Dis..

[107] Kim D.K., Zhang L., Dzau V.J., Pratt R.E. (1994). *H19*, a developmentally regulated gene, is reexpressed in rat vascular smooth muscle cells after injury. J. Clin. Investig..

[108] Han D.K., Khaing Z.Z., Pollock R.A., Haudenschild C.C., Liau G. (1996). H19, a marker of developmental transition, is reexpressed in human atherosclerotic plaques and is regulated by the insulin family of growth factors in cultured rabbit smooth muscle cells. J. Clin. Investig..

[109] Devlin A.M., Bottiglieri T., Domann F.E., Lentz S.R. (2005). Tissue-specific changes in H19 methylation and expression in mice with hyperhomocysteinemia. J. Biol. Chem..

[110] Lee J.H., Gao C., Peng G., Greer C., Ren S., Wang Y., Xiao X. (2011). Analysis of transcriptome complexity through RNA sequencing in normal and failing murine hearts. Circ. Res..

[111] Qin D.N., Qian L., Hu D.L., Yu Z.B., Han S.P., Zhu C., Wang X., Hu X. (2013). Effects of miR-19b overexpression on proliferation, differentiation, apoptosis and Wnt/β-catenin signaling pathway in P19 cell model of cardiac differentiation in vitro. Cell Biochem. Biophys..

[112] Han Y., Xu H., Cheng J., Zhang Y., Gao C., Fan T., Peng B., Li B., Liu L., Cheng Z. (2016). Downregulation of long non-coding RNA H19 promotes P19CL6 cells proliferation and inhibits apoptosis during late-stage cardiac differentiation via miR-19b-modulated Sox6. Cell Biosci..

[113] Tao H., Cao W., Yang J.J., Shi K.H., Zhou X., Liu L.P., Li J. (2016). Long noncoding RNA H19 controls DUSP5/ERK1/2 axis in cardiac fibroblast proliferation and fibrosis. Cardiovasc. Pathol..

[114] Holmes D.A., Yeh J.H., Yan D., Xu M., Chan A.C. (2015). Dusp5 negatively regulates IL-33-mediated eosinophil survival and function. EMBO J..

[115] Pan J.X. (2017). LncRNA H19 promotes atherosclerosis by regulating MAPK and NF-kB signaling pathway. Eur. Rev. Med. Pharmacol. Sci..

[116] Cai Y., Yang Y., Chen X., He D., Zhang X., Wen X., Hu J., Fu C., Qiu D., Jose P.A. (2016). Circulating “LncPPARδ” From Monocytes as a Novel Biomarker for Coronary Artery Diseases. Medicine.

[117] Ehrenborg E., Skogsberg J. (2013). Peroxisome proliferator-activated receptor delta and cardiovascular disease. Atherosclerosis.

[118] Han Y., Yang Y.N., Yuan H.H., Zhang T.T., Sui H., Wei X.L., Liu L., Huang P., Zhang W.J., Bai Y.X. (2014). UCA1, a long non-coding RNA up-regulated in colorectal cancer influences cell proliferation, apoptosis and cell cycle distribution. Pathology.

[119] Cheng N., Cai W., Ren S., Li X., Wang Q., Pan H., Zhao M., Li J., Zhang Y., Zhao C. (2015). Long non-coding RNA UCA1 induces non-T790M acquired resistance to EGFR-TKIs by activating the AKT/mTOR pathway in EGFR-mutant non-small cell lung cancer. Oncotarget.

[120] Wang X., Peng F., Cheng L., Yang G., Zhang D., Liu J., Chen X., Zhao S. (2017). Prognostic and clinicopathological role of long non-coding RNA UCA1 in various carcinomas. Oncotarget.

[121] Liu Y., Zhou D., Li G., Ming X., Tu Y., Tian J., Lu H., Yu B. (2015). Long non coding RNA-UCA1 contributes to cardiomyocyte apoptosis by suppression of p27 expression. Cell. Physiol. Biochem..

[122] Yan Y., Zhang B., Liu N., Qi C., Xiao Y., Tian X., Li T., Liu B. (2016). Circulating long noncoding RNA UCA1 as a novel biomarker of acute myocardial infarction. Biomed. Res. Int..

[123] Hrdlickova B., Kumar V., Kanduri K., Zhernakova D.V., Tripathi S., Karjalainen J., Lund R.J., Li Y., Ullah U., Modderman R. (2014). Expression profiles of long non-coding RNAs located in autoimmune disease-associated regions reveal immune cell-type specificity. Genome Med..

[124] Zemmour D., Pratama A., Loughhead S.M., Mathis D., Benoist C. (2017). Flicr, a long noncoding RNA, modulates Foxp3 expression and autoimmunity. Proc. Natl. Acad. Sci. USA.

[125] Mayama T., Marr A.K., Kino T. (2016). Differential expression of glucocorticoid receptor noncoding RNA repressor Gas5 in autoimmune and inflammatory diseases. Horm. Metab. Res..

[126] Firestein G.S. (2003). Evolving concepts of rheumatoid arthritis. Nature.

[127] Stuhlmüller B., Kunisch E., Franz J., Martinez-Gamboa L., Hernandez M.M., Pruss A., Ulbrich N., Erdmann V.A., Burmester G.R., Kinne R.W. (2003). Detection of oncofetal h19 RNA in rheumatoid arthritis synovial tissue. Am. J. Pathol..

[128] Song J., Kim D., Han J., Kim Y., Lee M., Jin E.J. (2015). PBMC and exosome-derived Hotair is a critical regulator and potent marker for rheumatoid arthritis. Clin. Exp. Med..

[129] Messemaker T.C., Frank-Bertoncelj M., Marques R.B., Adriaans A., Bakker A.M., Daha N., Gay S., Huizinga T.W., Toes R.E., Mikkers H.M. (2016). A novel long non-coding RNA in the rheumatoid arthritis risk locus TRAF1-C5 influences C5 mRNA levels. Genes Immun..

[130] Lu M.C., Yu H.C., Yu C.L., Huang H.B., Koo M., Tung C.H., Lai N.S. (2016). Increased expression of long noncoding RNAs LOC100652951 and LOC100506036 in T cells from patients with rheumatoid arthritis facilitates the inflammatory responses. Immunol. Res..

[131] Pan F., Zhu L., Lv H., Pei C. (2016). Quercetin promotes the apoptosis of fibroblast-like synoviocytes in rheumatoid arthritis by upregulating lncRNA MALAT1. Int. J. Mol. Med..

[132] Motterle A., Gattesco S., Caille D., Meda P., Regazzi R. (2015). Involvement of long non-coding RNAs in β cell failure at the onset of type 1 diabetes in NOD mice. Diabetologia.

[133] Sun C., Xue L., Zhu Z., Zhang F., Yang R., Yuan X., Jia Z., Liu Q. (2016). Insights from lncRNAs profiling of MIN6 β cells undergoing inflammation. Mediat. Inflamm..

[134] Rahman A., Isenberg D.A. (2008). Systemic lupus erythematosus. N. Engl. J. Med..

[135] Shi L., Zhang Z., Yu A.M., Wang W., Wei Z., Akhter E., Maurer K., Costa Reis P., Song L., Petri M. (2014). The SLE transcriptome exhibits evidence of chronic endotoxin exposure and has widespread dysregulation of non-coding and coding RNAs. PLoS ONE.

[136] Wu Y., Zhang F., Ma J., Zhang X., Wu L., Qu B., Xia S., Chen S., Tang Y., Shen N. (2015). Association of large intergenic noncoding RNA expression with disease activity and organ damage in systemic lupus erythematosus. Arthritis Res. Ther..

[137] Zhang F., Wu L., Qian J., Qu B., Xia S., La T., Wu Y., Ma J., Zeng J., Guo Q. (2016). Identification of the long noncoding RNA NEAT1 as a novel inflammatory regulator acting through MAPK pathway in human lupus. J. Autoimmun..

[138] Szegedi K., Sonkoly E., Nagy N., Németh I.B., Bata-Csörgo Z., Kemény L., Dobozy A., Széll M. (2010). The anti-apoptotic protein G1P3 is overexpressed in psoriasis and regulated by the non-coding RNA, PRINS. Exp. Dermatol..

[139] Sonkoly E., Bata-Csorgo Z., Pivarcsi A., Polyanka H., Kenderessy-Szabo A., Molnar G., Szentpali K., Bari L., Megyeri K., Mandi Y. (2005). Identification and characterization of a novel, psoriasis susceptibility-related noncoding RNA gene, PRINS. J. Biol. Chem..

[140] Szegedi K., Göblös A., Bacsa S., Antal M., Németh I.B., Bata-Csörgő Z., Kemény L., Dobozy A., Széll M. (2012). Expression and functional studies on the noncoding RNA, PRINS. Int. J. Mol. Sci..

[141] Holm S.J., Sánchez F., Carlén L.M., Mallbris L., Ståhle M., O’Brien K.P. (2005). HLA-Cw*0602 associates more strongly to psoriasis in the Swedish population than variants of the novel 6p21.3 gene *PSORS1C3*. Acta Derm. Venereol..

[142] Chang Y.T., Chou C.T., Shiao Y.M., Lin M.W., Yu C.W., Chen C.C., Huang C.H., Lee D.D., Liu H.N., Wang W.J. (2006). Psoriasis vulgaris in Chinese individuals is associated with *PSORS1C3* and *CDSN* genes. Br. J. Dermatol..

[143] Li B., Tsoi L.C., Swindell W.R., Gudjonsson J.E., Tejasvi T., Johnston A., Ding J., Stuart P.E., Xing X., Kochkodan J.J. (2014). Transcriptome analysis of psoriasis in a large case-control sample: RNA-seq provides insights into disease mechanisms. J. Investig. Dermatol..

[144] Tsoi L.C., Iyer M.K., Stuart P.E., Swindell W.R., Gudjonsson J.E., Tejasvi T., Sarkar M.K., Li B., Ding J., Voorhees J.J. (2015). Analysis of long non-coding RNAs highlights tissue-specific expression patterns and epigenetic profiles in normal and psoriatic skin. Genome Biol..

[145] Ahn R., Gupta R., Lai K., Chopra N., Arron S.T., Liao W. (2016). Network analysis of psoriasis reveals biological pathways and roles for coding and long non-coding RNAs. BMC Genom..

[146] Gupta R., Ahn R., Lai K., Mullins E., Debbaneh M., Dimon M., Arron S., Liao W. (2016). Landscape of long noncoding RNAs in psoriatic and healthy skin. J. Investig. Dermatol..

[147] Wang J., Peng H., Tian J., Ma J., Tang X., Rui K., Tian X., Wang Y., Chen J., Lu L. (2016). Upregulation of long noncoding RNA TMEVPG1 enhances T helper type 1 cell response in patients with Sjögren syndrome. Immunol. Res..

[148] Jiang C., Fang X., Jiang Y., Shen F., Hu Z., Li X., Huang X. (2016). TNF-α induces vascular endothelial cells apoptosis through overexpressing pregnancy induced noncoding RNA in Kawasaki disease model. Int. J. Biochem. Cell Biol..

[149] Li Z., Chao T.C., Chang K.Y., Lin N., Patil V.S., Shimizu C., Head S.R., Burns J.C., Rana T.M. (2014). The long noncoding RNA THRIL regulates TNF-α expression through its interaction with hnRNPL. Proc. Natl. Acad. Sci. USA.

[150] Shirasawa S., Harada H., Furugaki K., Akamizu T., Ishikawa N., Ito K., Tamai H., Kuma K., Kubota S., Hiratani H. (2004). SNPs in the promoter of a B cell-specific antisense transcript, SAS-ZFAT, determine susceptibility to autoimmune thyroid disease. Hum. Mol. Genet..

[151] Castellanos-Rubio A., Fernandez-Jimenez N., Kratchmarov R., Luo X., Bhagat G., Green P.H., Schneider R., Kiledjian M., Bilbao J.R., Ghosh S. (2016). A long noncoding RNA associated with susceptibility to celiac disease. Science.

[152] Doyle K.M., Kennedy D., Gorman A.M., Gupta S., Healy S.J., Samali A. (2011). Unfolded proteins and endoplasmic reticulum stress in neurodegenerative disorders. J. Cell. Mol. Med..

[153] Taylor J.P., Hardy J., Fischbeck K.H. (2002). Toxic proteins in neurodegenerative disease. Science.

[154] Ransohoff R.M. (2016). How neuroinflammation contributes to neurodegeneration. Science.

[155] Irvine G.B., El-Agnaf O.M., Shankar G.M., Walsh D.M. (2008). Protein aggregation in the brain: The molecular basis for Alzheimer’s and Parkinson’s diseases. Mol. Med..

[156] Corder E.H., Saunders A.M., Strittmatter W.J., Schmechel D.E., Gaskell P.C., Small G.W., Roses A.D., Haines J.L., Pericak-Vance M.A. (1993). Gene dose of apolipoprotein E type 4 allele and the risk of Alzheimer’s disease in late onset families. Science.

[157] Faghihi M.A., Modarresi F., Khalil A.M., Wood D.E., Sahagan B.G., Morgan T.E., Finch C.E., St Laurent G., Kenny P.J., Wahlestedt C. (2008). Expression of a noncoding RNA is elevated in Alzheimer’s disease and drives rapid feed-forward regulation of beta-secretase. Nat. Med..

[158] Liu T., Huang Y., Chen J., Chi H., Yu Z., Wang J., Chen C. (2014). Attenuated ability of BACE1 to cleave the amyloid precursor protein via silencing long noncoding RNA BACE1-AS expression. Mol. Med. Rep..

[159] Massone S., Vassallo I., Fiorino G., Castelnuovo M., Barbieri F., Borghi R., Tabaton M., Robello M., Gatta E., Russo C. (2011). 17A, a novel non-coding RNA, regulates GABA B alternative splicing and signaling in response to inflammatory stimuli and in Alzheimer disease. Neurobiol. Dis..

[160] Zhou X., Xu J. (2015). Identification of Alzheimer’s disease-associated long noncoding RNAs. Neurobiol. Aging.

[161] Magistri M., Velmeshev D., Makhmutova M., Faghihi M.A. (2015). Transcriptomics profiling of Alzheimer’s disease reveal neurovascular defects, altered amyloid-β homeostasis, and deregulated expression of long noncoding RNAs. J. Alzheimers Dis..

[162] Polymeropoulos M.H., Lavedan C., Leroy E., Ide S.E., Dehejia A., Dutra A., Pike B., Root H., Rubenstein J., Boyer R. (1997). Mutation in the α-synuclein gene identified in families with Parkinson’s disease. Science.

[163] Chaudhuri K.R., Healy D.G., Schapira A.H., Excellence N.I.F.C. (2006). Non-motor symptoms of Parkinson’s disease: Diagnosis and management. Lancet Neurol..

[164] Lesage S., Brice A. (2009). Parkinson’s disease: From monogenic forms to genetic susceptibility factors. Hum. Mol. Genet..

[165] Zhang Q.S., Wang Z.H., Zhang J.L., Duan Y.L., Li G.F., Zheng D.L. (2016). β-Asarone protects against MPTP-induced Parkinson’s disease via regulating long non-coding RNA MALAT1 and inhibiting α-synuclein protein expression. Biomed. Pharmacother..

[166] Kraus T.F.J., Haider M., Spanner J., Steinmaurer M., Dietinger V., Kretzschmar H.A. (2017). Altered long noncoding RNA expression precedes the course of Parkinson’s disease—A Preliminary Report. Mol. Neurobiol..

[167] Ni Y., Huang H., Chen Y., Cao M., Zhou H., Zhang Y. (2017). Investigation of long non-coding RNA expression profiles in the substantia nigra of Parkinson’s disease. Cell. Mol. Neurobiol..

[168] Cookson M.R. (2010). The role of leucine-rich repeat kinase 2 (LRRK2) in Parkinson’s disease. Nat. Rev. Neurosci..

[169] Hossein-Nezhad A., Fatemi R.P., Ahmad R., Peskind E.R., Zabetian C.P., Hu S.C., Shi M., Wahlestedt C., Zhang J., Faghihi M.A. (2016). Transcriptomic profiling of extracellular RNAs present in cerebrospinal fluid identifies differentially expressed transcripts in Parkinson’s disease. J. Parkinsons Dis..

[170] Wang S., Zhang X., Guo Y., Rong H., Liu T. (2017). The long noncoding RNA HOTAIR promotes Parkinson’s disease by upregulating LRRK2 expression. Oncotarget.

[171] Johnson R., Richter N., Jauch R., Gaughwin P.M., Zuccato C., Cattaneo E., Stanton L.W. (2010). Human accelerated region 1 noncoding RNA is repressed by REST in Huntington’s disease. Physiol. Genom..

[172] Francelle L., Galvan L., Gaillard M.C., Petit F., Bernay B., Guillermier M., Bonvento G., Dufour N., Elalouf J.M., Hantraye P. (2015). Striatal long noncoding RNA Abhd11os is neuroprotective against an N-terminal fragment of mutant huntingtin in vivo. Neurobiol. Aging.

[173] Nishimoto Y., Nakagawa S., Hirose T., Okano H.J., Takao M., Shibata S., Suyama S., Kuwako K., Imai T., Murayama S. (2013). The long non-coding RNA nuclear-enriched abundant transcript 1_2 induces paraspeckle formation in the motor neuron during the early phase of amyotrophic lateral sclerosis. Mol. Brain.

[174] Huang X., Luo Y.L., Mao Y.S., Ji J.L. (2017). The link between long noncoding RNAs and depression. Prog. Neuropsychopharmacol. Biol. Psychiatry.

[175] Chubb J.E., Bradshaw N.J., Soares D.C., Porteous D.J., Millar J.K. (2008). The DISC locus in psychiatric illness. Mol. Psychiatry.

[176] Millar J.K., Wilson-Annan J.C., Anderson S., Christie S., Taylor M.S., Semple C.A., Devon R.S., St Clair D.M., Muir W.J., Blackwood D.H. (2000). Disruption of two novel genes by a translocation co-segregating with schizophrenia. Hum. Mol. Genet..

[177] Barry G., Briggs J.A., Vanichkina D.P., Poth E.M., Beveridge N.J., Ratnu V.S., Nayler S.P., Nones K., Hu J., Bredy T.W. (2014). The long non-coding RNA Gomafu is acutely regulated in response to neuronal activation and involved in schizophrenia-associated alternative splicing. Mol. Psychiatry.

[178] Liao J., He Q., Li M., Chen Y., Liu Y., Wang J. (2016). LncRNA MIAT: Myocardial infarction associated and more. Gene.

[179] Rao S.Q., Hu H.L., Ye N., Shen Y., Xu Q. (2015). Genetic variants in long non-coding RNA MIAT contribute to risk of paranoid schizophrenia in a Chinese Han population. Schizophr. Res..

[180] Carter M.T., Nikkel S.M., Fernandez B.A., Marshall C.R., Noor A., Lionel A.C., Prasad A., Pinto D., Joseph-George A.M., Noakes C. (2011). Hemizygous deletions on chromosome 1p21.3 involving the *DPYD* gene in individuals with autism spectrum disorder. Clin. Genet..

[181] Xu B., Ionita-Laza I., Roos J.L., Boone B., Woodrick S., Sun Y., Levy S., Gogos J.A., Karayiorgou M. (2012). De novo gene mutations highlight patterns of genetic and neural complexity in schizophrenia. Nat. Genet..

[182] Gianfrancesco O., Warburton A., Collier D.A., Bubb V.J., Quinn J.P. (2017). Novel brain expressed RNA identified at the MIR137 schizophrenia-associated locus. Schizophr. Res..

[183] Steen V.M., Nepal C., Ersland K.M., Holdhus R., Nævdal M., Ratvik S.M., Skrede S., Håvik B. (2013). Neuropsychological deficits in mice depleted of the schizophrenia susceptibility gene CSMD1. PLoS ONE.

[184] Cui X., Niu W., Kong L., He M., Jiang K., Chen S., Zhong A., Zhang Q., Li W., Lu J. (2017). Long noncoding RNA as an indicator differentiating schizophrenia from major depressive disorder and generalized anxiety disorder in nonpsychiatric hospital. Biomark. Med..

[185] Chen S., Sun X., Niu W., Kong L., He M., Li W., Zhong A., Lu J., Zhang L. (2016). Aberrant expression of long non-coding RNAs in schizophrenia patients. Med. Sci. Monit..

[186] Spadaro P.A., Flavell C.R., Widagdo J., Ratnu V.S., Troup M., Ragan C., Mattick J.S., Bredy T.W. (2015). Long noncoding RNA-directed epigenetic regulation of gene expression is associated with anxiety-like behavior in mice. Biol. Psychiatry.

[187] Murphy T.M., Crawford B., Dempster E.L., Hannon E., Burrage J., Turecki G., Kaminsky Z., Mill J. (2017). Methylomic profiling of cortex samples from completed suicide cases implicates a role for PSORS1C3 in major depression and suicide. Transl. Psychiatry.

[188] Gupta M.A., Pur D.R., Vujcic B., Gupta A.K. (2017). Suicidal behaviors in the dermatology patient. Clin. Dermatol..

[189] Ye N., Rao S., Du T., Hu H., Liu Z., Shen Y., Xu Q. (2017). Intergenic variants may predispose to major depression disorder through regulation of long non-coding RNA expression. Gene.

[190] Modarresi F., Faghihi M.A., Lopez-Toledano M.A., Fatemi R.P., Magistri M., Brothers S.P., van der Brug M.P., Wahlestedt C. (2012). Inhibition of natural antisense transcripts in vivo results in gene-specific transcriptional upregulation. Nat. Biotechnol..

[191] Bahi A., Chandrasekar V., Dreyer J.L. (2014). Selective lentiviral-mediated suppression of microRNA124a in the hippocampus evokes antidepressants-like effects in rats. Psychoneuroendocrinology.

[192] Kocerha J., Dwivedi Y., Brennand K.J. (2015). Noncoding RNAs and neurobehavioral mechanisms in psychiatric disease. Mol. Psychiatry.

[193] Liu Z., Li X., Sun N., Xu Y., Meng Y., Yang C., Wang Y., Zhang K. (2014). Microarray profiling and co-expression network analysis of circulating lncRNAs and mRNAs associated with major depressive disorder. PLoS ONE.

[194] Mus E., Hof P.R., Tiedge H. (2007). Dendritic BC200 RNA in aging and in Alzheimer’s disease. Proc. Natl. Acad. Sci. USA.

[195] Kerin T., Ramanathan A., Rivas K., Grepo N., Coetzee G.A., Campbell D.B. (2012). A noncoding RNA antisense to moesin at 5p14.1 in autism. Sci. Transl. Med..

[196] DeWitt J.J., Grepo N., Wilkinson B., Evgrafov O.V., Knowles J.A., Campbell D.B. (2016). Impact of the autism-associated long noncoding RNA MSNP1AS on neuronal architecture and gene expression in human neural progenitor cells. Genes.

[197] Williams J.M., Beck T.F., Pearson D.M., Proud M.B., Cheung S.W., Scott D.A. (2009). A 1q42 deletion involving DISC1, DISC2, and TSNAX in an autism spectrum disorder. Am. J. Med. Genet. A.

[198] Noor A., Whibley A., Marshall C.R., Gianakopoulos P.J., Piton A., Carson A.R., Orlic-Milacic M., Lionel A.C., Sato D., Pinto D. (2010). Disruption at the PTCHD1 locus on Xp22.11 in autism spectrum disorder and intellectual disability. Sci. Transl. Med..

[199] Ziats M.N., Rennert O.M. (2013). Aberrant expression of long noncoding RNAs in autistic brain. J. Mol. Neurosci..

[200] Wang Y., Zhao X., Ju W., Flory M., Zhong J., Jiang S., Wang P., Dong X., Tao X., Chen Q. (2015). Genome-wide differential expression of synaptic long noncoding RNAs in autism spectrum disorder. Transl. Psychiatry.

